# Construction and sequencing of an infectious clone of the goose embryo-adapted Muscovy duck parvovirus vaccine strain FZ91-30

**DOI:** 10.1186/s12985-016-0564-9

**Published:** 2016-06-21

**Authors:** Jianye Wang, Yu Huang, Mingxu Zhou, Philip R. Hardwidge, Guoqiang Zhu

**Affiliations:** College of Veterinary Medicine, Yangzhou University, 48 Wenhui East Road, 225009 Yangzhou, Peoples’ Republic of China; College of Veterinary Medicine, Kansas State University, Manhattan, KS USA

**Keywords:** Muscovy duck parvovirus, Attenuation, Infectious clone, Rescue, Transfection

## Abstract

**Background:**

Muscovy duck parvovirus (MDPV) is the etiological agent of Muscovy duckling parvoviral disease, which is characterized by diarrhea, locomotive dysfunction, stunting, and death in young ducklings, and causes substantial economic losses in the Muscovy duck industry worldwide. FZ91-30 is an attenuated vaccine strain that is safe and immunogenic to ducklings, but the genomic information and molecular mechanism underlining the attenuation are not understood.

**Methods:**

The FZ91-30 strain was propagated in 11-day-old embryonated goose eggs, and viral particles were purified from the pooled allantoic fluid by differential centrifugation and ultracentrifugation. Single-stranded genomic DNA was extracted and annealed to form double-stranded DNA. The dsDNA digested with *Nco*I resulted two sub-genomic fragments, which were then cloned into the modified plasmid pBluescript II SK, respectively, generating plasmid pBSKNL and pBSKNR. The sub-genomic plasmid clones were sequenced and further combined to construct the plasmid pFZ that contained the entire genome of strain FZ91-30. The complete genome sequences of strain FM and YY and partial genome sequences of other strains were retrieved from GenBank for sequence comparison. The plasmid pFZ containing the entire genome of FZ91-30 was transfected in 11-day-old embryonated goose eggs via the chorioallantoic membranes route to rescue infectious virus. A genetic marker was introduced into the rescued virus to discriminate from its parental virus.

**Results:**

The genome of FZ91-30 consists of 5,131 nucleotides and has 98.9 % similarity to the FM strain. The inverted terminal repeats (ITR) are 456 nucleotides in length, 14 nucleotides longer than that of Goose parvovirus (GPV). The exterior 415 nucleotides of the ITR form a hairpin structure, and the interior 41 nucleotides constitute the D sequence, a reverse complement of the D′ sequence at the 3′ ITR. Amino acid sequence alignment of the VP1 proteins between FZ91-30 and five pathogenic MDPV strains revealed that FZ91-30 had five mutations; two in the unique region of the VP1 protein (VP1u) and three in VP3. Sequence alignment of the Rep1 proteins revealed two amino acid alterations for FZ91-30, both of which were conserved for two pathogenic strains YY and P. Transfection of the plasmid pFZ in 11-day-old embryonated goose eggs resulted in generation of infectious virus with similar biological properties as compared with the parental strain.

**Conclusions:**

The amino acid mutations identified in the VP1 and Rep1 protein may contribute to the attenuation of FZ91-30 in Muscovy ducklings. Plasmid transfection in embryonated goose eggs was suitable for rescue of infectious MDPV.

## Background

Muscovy duck parvovirus (MDPV) belongs to the *Dependoparvovirus* genus in the *Parvoviridae* family. Its closest relative is Goose parvovirus (GPV) [1]. MDPV is the etiological agent of Muscovy duckling parvoviral disease [2–5], which is also named “three-week disease” in China [6]. The disease is characterized by diarrhea, locomotive dysfunction, stunting, and death in young ducklings [2, 7], and causes substantial economic losses in the Muscovy duck industry worldwide.

The MDPV genome is a linear, single-stranded DNA molecule of ~5.1 kb [8]. The genome is flanked by inverted terminal repeats (ITR; 457 nts in the FM strain) that can form a palindromic hairpin structure to serve as origin of replication [9]. The ITR also contains *cis*-acting elements required for rescue, excision from cloning vectors, and packaging [10].

The left open reading frame encodes the non-structural protein Rep1 as well as several smaller proteins generated after splicing [11, 12]. Rep proteins can bind to ITR elements and are involved in genome replication, and modulation of the downstream P41 promoter [13, 14]. The right open reading frame encodes the capsid proteins VP1, VP2, and VP3 that are generated from the use of different initiation codons and through proteolytic cleavage. VP1, VP2, and VP3 comprise the viral capsid at a ratio of ~1:1:8 [8].

Live vaccines have been utilized in day-old Muscovy ducklings to prevent disease outbreaks. These attenuated vaccines were prepared by serial passages of the parental derivatives in cell cultures or embryonated eggs [15–17]. FZ91 is a virulent isolate obtained from deceased ducklings that died from an outbreak of “three-week” disease in a farm from Fuzhou city, China [18]. FZ91 was passaged 3 times in embryonated Muscovy eggs and in embryonated goose eggs 30 times, resulting in an attenuated virus that was safe and immunogenic to ducklings. To discriminate the vaccine strain from its parental strain FZ91, the attenuated virus is referred to as FZ91-30.

Here we sequenced the FZ91-30 genome and generated an infectious plasmid clone. We found that several amino acid differences between FZ91-30 and virulent isolates may contribute to MDPV attenuation.

## Results

### Genome sequence analysis

**Fig. 1:**
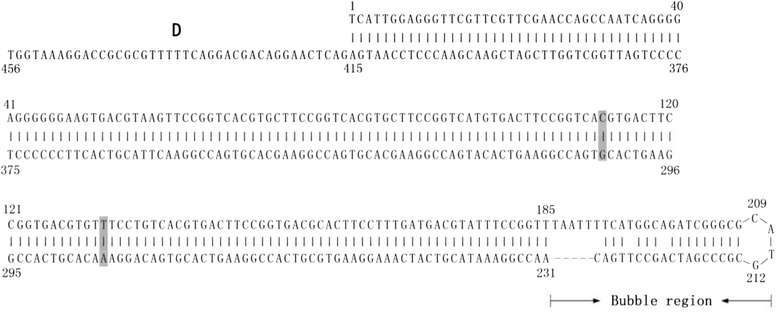
FZ91-30 5′ inverted terminal repeat (ITR) sequences. The numbers above the sequences denote the nucleotide position. The nucleotide differences relative to the FM strain are shaded with grey. The D sequence is composed of 42 nucleotides, which base pairs with the D′ sequence at the 3′ ITR. The sequences in the bubble regions can exist as two different sequences, termed flip and flop, which reversely complement with each other

We assembled the complete genome of FZ91-30 after sequencing the sub-genomic fragments. The complete genome is comprised of 5,131 nucleotides and has 98.9 % similarity to the FM strain, with the latter having an additional “C” nucleotide at the 5′ terminus of the genome. The ITR of FZ91-30 consists of 456 nucleotides, which forms the palindromic structure at the 5′ and 3′ termini. 370 nucleotides are involved in base-pairing in the stem region of the palindrome, and 45 nucleotides constitute the bubble region. The bubble region (45 nts) has two different sequences in the plus-stranded genome, named flip and flop, which form reverse complements. In comparison to the FM strain, FZ91-30 had 4 nucleotide changes in the ITR stems, but at base-pairing positions that did not alter the hairpin structure (Fig. 1). The interior of the ITR is a region designated as the D sequence, comprised of 41 nucleotides. The D sequence at the 5′ ITR reversely complements with the D′ sequence at the 3′ ITR.

### VP1 and Rep1 amino acid sequences comparisons

**Fig. 2:**
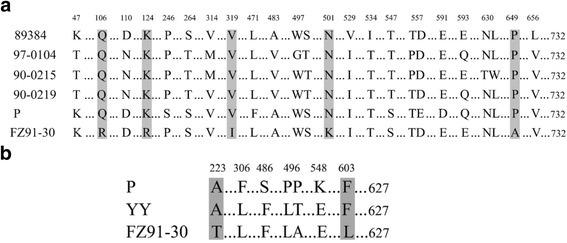
Amino acid sequence alignments of the VP1 proteins (**a**) and the Rep1 proteins (**b**) between the FZ91-30 and other pathogenic isolates. Amino acids in common are indicated by *dots*. Different amino acids are denoted by the numbers above the alignments and shaded in *grey*

The VP1 protein of FZ91-30 displayed 98.0 ~ 98.8 % homology at the amino acid level with five pathogenic isolates, 97.8 % homology with the FM strain, and had the highest homology (98.8 %) with the 89384 strain from France. Amino acid sequence alignments of the VP1 proteins revealed that FZ91-30 had a total of five amino acid differences in comparison to the five pathogenic isolates (89384, 97-0104, 90-0215, 90-0219 and P) (Fig. 2a). Two differences (Q106R and K124R) were in the unique region of the VP1 protein (VP1u), and three variations (V319I, N501K, and P649A) occurred in the VP3 protein. The Rep1 protein of F91-30 displayed 99.5 % and 98.9 % homology at the amino acid level with two pathogenic strain YY and P, respectively. Sequence alignment of the Rep1 proteins revealed that FZ91-30 had produced two amino acid mutations (A223T and F603L), both of which were conserved for strain YY and P (Fig. 2b). These amino acid differences in VP1 and Rep1 proteins may contribute the attenuation of FZ91-30 after serial passages in embryonated goose eggs.

### Characterization and rescue of pFZ

**Fig. 3:**
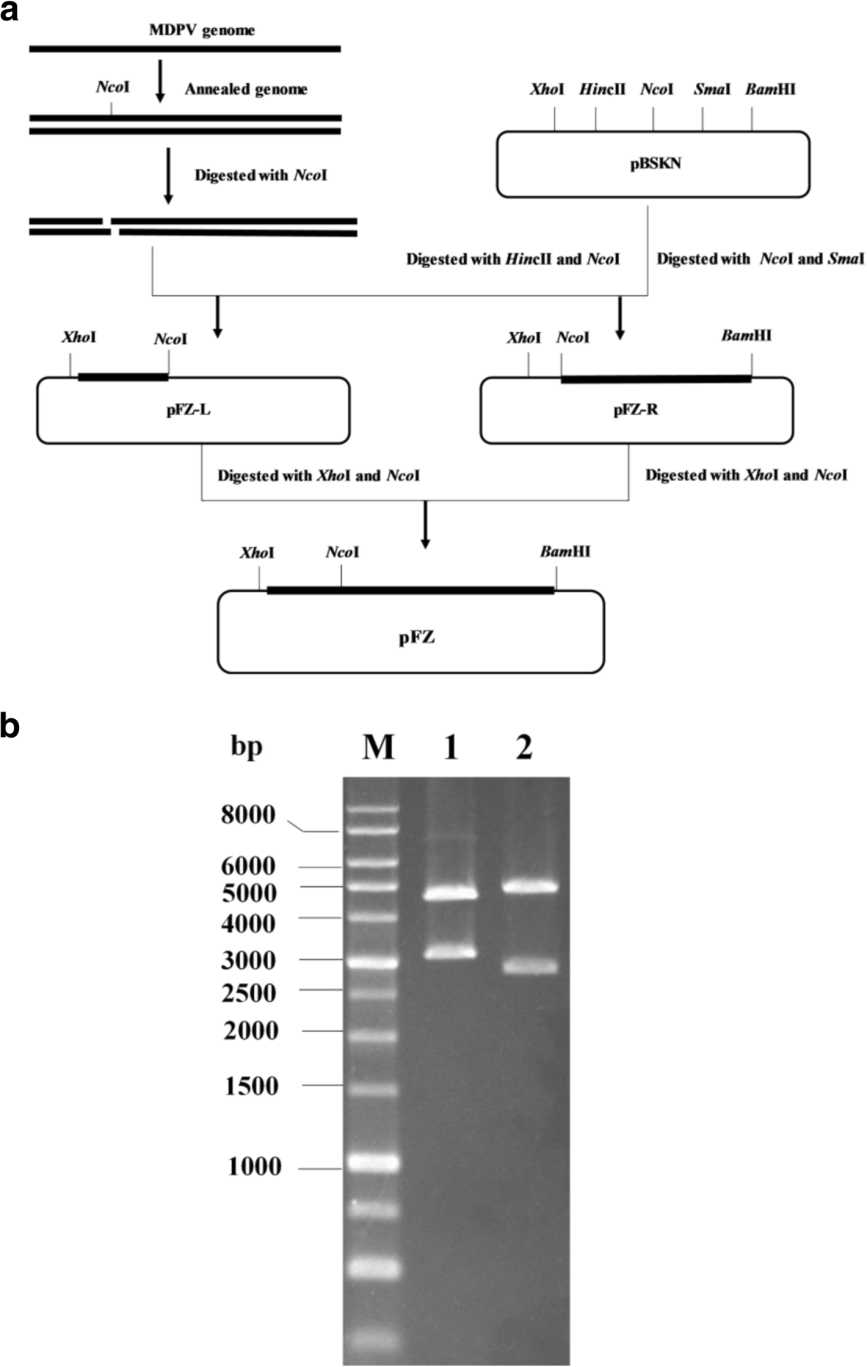
Experimental strategy used to construct the infectious FZ91-30 MDPV plasmid clone. **a** The digested MDPV subgenomic fragments were cloned into the pBSKN plasmid respectively and further combined to form the recombinant plasmid pFZ that contained the whole genome of FZ91-30. **b** Restriction enzyme digestion of pFZ. M: DNA markers. 1: Digestion with *Sph*I. 2: Double digestion with *Xho*I and *Bam*HI

With combination of the cloned sub-genomic fragments, the FZ91-30 genome was cloned into the pBluescript II SK vector (Agilent, Santa Clara, USA), resulting in generation of the plasmid pFZ (Fig. 3a). Since the genome of FZ91-30 lacks *Bam*HI and *Xho*I sites, digestion of pFZ with *Bam*HI and *Xho*I produced a 5.1-kb genomic molecule and a 2.9-kb vector molecule. Since there are two *Sph*I sites distributed in the ITRs, digestion of pFZ with *Sph*I produced 3.3-kb and 4.7-kb DNA fragments (Fig. 3b).

**Fig. 4:**
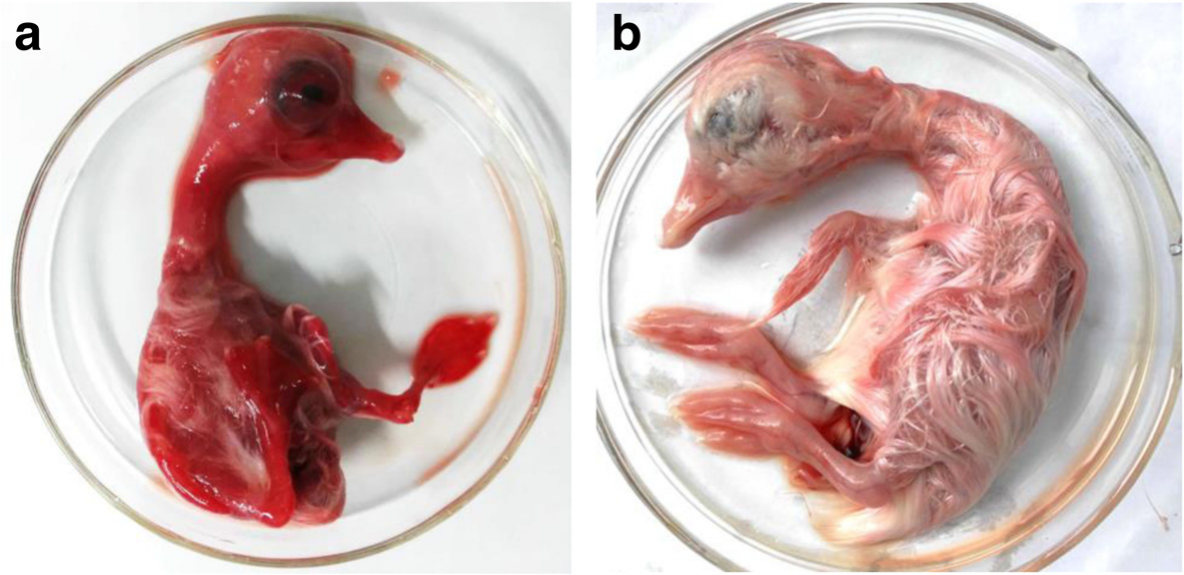
Pathogenic changes of the embryo died at day 7 post-transfection of pFZ (**a**), and haemorrhagical lesions in the head, neck, embryonic body, and legs were observed. In the control group (**b**), no pathogenic change was observed in the embryo which survived in transfection of the vector plasmid pBSKN till the 12th day

Embryos began to die 7 days after their transfection with pFZ and mortality rates reached 70 % at day 12 post-transfection. The allantoic fluid was pooled for further passages in 11-day-old embryonated goose eggs. The rescued virus were successfully passaged in embryonated goose eggs four times and killed the embryos between 96 h and 192 h. The dead embryos showed pathogenic lesions typical of MDPV infection, including hemorrhagic embryos (Fig. 4a) and edematous chorioallantoic membranes. In the control group, all of five goose embryos survived after transfection of the vector plasmid pBSKN during the observation period. Examination of these embryos revealed no obvious pathogenic change (Fig. 4b).

### Discrimination of the rescued virus from its parental strain

**Fig. 5:**
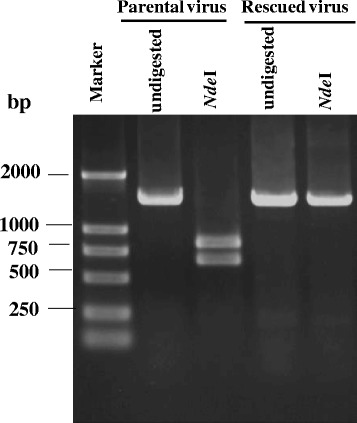
Discrimination of the rescued virus from the parental strain FZ91-30 using *Nde*I digestion

A single nucleotide mutation was introduced into the rescued virus. A 1.5-kb DNA fragment covering the mutation site was amplified from the rescued virus. The amplicon from the rescued virus could not be cut with *Nde*I, while the amplicon from the parental virus could be cut with *Nde*I, resulting in a 0.7-kb and a 0.8-kb fragment (Fig. 5). The result showed that the rescued virus originated from the transfected plasmid pFZ, eliminating the contamination of the parental virus during viral passages. Sequencing of the whole genome of the rescued virus showed that it had an identical genome sequence to the parental strain FZ91-30, except for the introduced nucleotide mutation in the Rep gene.

### Calculation of the ELD_50_ and MDT

The medium embryos lethal dose (ELD_50_) and mean death time (MDT) of the rescued virus in 11-day-old susceptible embryonated goose eggs was 5 × 10^3.5^/ml and 156 h, respectively, while the ELD_50_ and MDT of the parental strain FZ91-30 was 5 × 10^3.36^/ml and 159 h, respectively. Thus the rescued virus had similar viral titers and virulence in embryonated goose eggs as compared with the parental strain.

## Discussion

Among all the members in the *Parvoviridae* family, pathogenic waterfowl parvoviruses and the B19 parvovirus possess the longest ITR sequences [9, 19]. Although the genome of MDPV is only 5.1-kb long, the secondary structure in the ITR makes it difficult to clone the full-length genome with one-step PCR amplification or by direct genome cloning [9, 20]. It is feasible to clone the sub-genomic fragments respectively and then to combine the sub-genomic fragments to form the full-length genomic clone. We obtained the plus-stranded genome sequence of MDPV by sequencing several sub-genomic clones, except for the sequences in the bubble region at the 5' terminus and the 3' terminus. The sequences in the bubble region may exist in two opposite orientations, termed flip and flop, which form reverse complements. The unique *Sph*I site at the loop position of the bubble region allowed us to cut the entire ITR into two halves and then sequence each subclone containing half of the ITR.

The Sure strain is a kind of *E.coli* which is engineered for cloning certain DNA segments that are unstable in conventional *E. coli* strains. The Sure competent cells was used in the transformation experiment for sub-genome cloning. Our results herein demonstrated that all of positive plasmid clones from the Sure strain *E.coli* harbored the ITR sequences without deletions. Simultaneously, we also attempted to use the DH5α competent cells for transformation comparison with the Sure strain, revealing that all of the screened positive plasmid colonies propagated in DH5α *E.coli* contained ITR deletions in various degrees. However, a subset of recombinant plasmids containing the MDPV genome had partially deleted ITRs but were still infectious, as evidenced with successful viral rescue after plasmid transfection in 11-day-old embryonated goose eggs. The deleted portions in the ITRs could be repaired in rescued virions by PCR characterization, implying that MDPV might utilize a similar molecular mechanism as adopted by adeno-associated virus (AAV) for correcting its deleted ITRs in the course of viral rescue [21]. The phenomena also supports the current parvoviral classification at some extent that MDPV and GPV are classified into the *Dependoparvovirus* genus from the primary *Autonomous* genus [1].

The vaccine strain FZ91-30 is the 30th passage in embryonated goose eggs, which is nonpathogenic for 1-day-old Muscovy ducklings. Prior to our work, complete genome data for MDPV were very limited and most of the MDPV sequences corresponded to the VP1 genes and few sequences about the Rep1 gene were available. Therefore, sufficient VP1 protein sequences from the virulent strains of MDPV could be used for comparison with that of strain FZ91-30 in this study. Six amino acid mutations were identified in the vaccine strain, while these sites were conserved in all five virulent strains of MDPV. Parvoviral infection of the host depends on the interaction between the receptor and host cells [22, 23]. The binding domain of the receptor lies in the surface of the viral capsid and thus these mutated amino acids that arose during viral passage probably play a crucial role in the attenuation of FZ91-30. In the previous studies, the phospholipase A_2_ (PLA_2_) motifs and nuclear localization signals (NLS), which are important for parvoviral infection of host cells, are identified to mainly lie in the VP1 unique region (VP1u) [24, 25]. However, with careful amino acid sequence alignment between FZ91-30 and its counterparts in the *Parvoviridae* family*,* we found that both mutations (Q106R and K124R) in the VP1u of FZ91-30 did not reside in the scope of the identified PLA_2_ and NLS motifs, indicating that the crucial amino acid sites for the activities of PLA_2_ and NLS motifs were conserved for both virulent and attenuated strains of MDPV.

Our determination of the genome sequence and construction of an infectious clone of FZ91-30 provide a foundation for further studies of MDPV in Muscovy ducklings.

## Conclusions

The full-length genome sequence of strain FZ91-30 with 5131 nucleotides was determined. The amino acid mutations identified in the VP1 and Rep1 protein may contribute to the attenuation of FZ91-30 in Muscovy ducklings. Compared to the experimental procedure for viral rescue in cultured cells, plasmid transfection performed in embryonated goose eggs is a simple and efficacious method for rescue of infectious MDPV. Generation of the infectious plasmid pFZ may facilitate future studies of MDPV virulence.

## Methods

### Virus propagation and purification

FZ91-30 was diluted 1:50 in sterile saline containing penicillin (2,000 U/ml) and streptomycin (2,000 U/ml) and inoculated into the chorioallantoic cavity of 11-day-old embryonated goose eggs. Embryos died from MDPV infection after 48 h were kept at 4 °C for 6 h, and then their allantoic fluid was pooled. Approximately 300 ml of allantoic fluid was centrifuged at 11, 000 g for 20 min. Chloroform (1/2 *v/v*) was added to the supernatant, shaken intensively, and then subjected to centrifugation at 11, 000 g for 20 min. The upper aqueous phase containing virus was transferred and pelleted by ultracentrifugation at 150,000 g for 3 h (SW32Ti rotor, Beckman), and the virus-containing pellet was resuspended in 5 ml of TE buffer (50 mM Tris, 20 mM EDTA, pH 8.0).

### Viral DNA extraction

Viral DNA was extracted as previously described [26], dissolved in STE buffer (10 mM Tris, 1 mM EDTA, 100 mM NaCl, pH 8.0), and then annealed by heating to 95 °C for 5 min followed by slow cooling to 60 °C.

### Construction of the infectious clone

To facilitate cloning, the *Eco*RV site was replaced with *Nco*I (New England Biolabs, Ipswich, USA) in plasmid pBluescript II SK (Agilent, Santa Clara, USA), resulting in plasmid pBSKN. MDPV dsDNA was digested with *Nco*I, which cuts the genome at nucleotide position 723. The digested fragments were separated using electrophoresis and purified with a gel extraction kit (Tiangen, Peiking, China). The 0.7-kb fragment was ligated into the *Hin*cII-*Nco*I site of pBSKN, generating plasmid pBSKNL. The 4.4-kb fragment was ligated into the *Nco*I-*Sma*I site of pBSKN, generating plasmid pBSKNR (Fig. 3a). The ligated products were transformed into competent cells of the Sure strain {e14-(McrA-), Δ *(mcrCB-hsdSMR-mrr) 171 endA1 gyrA96 thi-1 supE44 relA1 lac recB recJ sbcC umuC*::Tn*5* (Kanr) *uvrC* [F’ *proAB lacI*q*Z*Δ*M15* Tn*10* (Tetr)]} (Agilent, Santa Clara, USA). pBSKNL was digested with *Xho*I and *Nco*I, thus generating a 0.7-kb fragment with *Xho*I/*Nco*I sites at either end. The 0.7-kb fragment was then ligated into *Xho*I/*Nco*I-digested pBSKNR, resulting in plasmid pFZ that contained the entire genome of the FZ91-30 strain.

### Sequence analysis and comparison

The cloned genomic fragments were sequenced using an ABI-PRISM3730 automated sequencer and BigDye terminators v3.1 (Applied Biosystems, Foster City, USA). To overcome the difficulties in sequencing the ITRs, the plasmids were digested with *Sph*I and *Bam*HI or with *Xho*I. *Sph*I cut the ITR in the middle loop position and the resulting fragments were sub-cloned into pUC19 or pBluescript II for sequencing. The sequences were assembled using SeqManII software included in the Lasergene package 5.0 (DNASTAR, Madison, USA). The complete genome sequence was submitted to GenBank, accession number KT865605.

For comparative studies, the complete MDPV genome sequences of strain FM and YY and the partial genome sequences of five pathogenic strains of MDPV (Table 1) were retrieved from GenBank. The hosts for three Taiwanese isolates (90-0215, 90-0219, and 97-0104) were Muscovy duck, mule duck, and goose, respectively [27]. 89384 was a France isolate from Muscovy duck [28]. P strain was isolated from Muscovy duck in mainland of China [29]. Sequence comparisons were performed using the MegAlign program packaged in the Lasergene package 5.0.

**Table 1:** MDPV isolates used in this study for sequence comparison

Strain	Pathogenicity	Geographic origin	Genome region	Year of isolation	GenBank accession no.
FM	n. a.	Europe	Full-length	n. a.	U22967
89384	Pathogenic	France	VP1	1989	Z68272
90-0215	Pathogenic	Taiwan	VP1	1990	AY382891
90-0219	Pathogenic	Taiwan	VP1	1990	AY382892
97-0104	Pathogenic	Taiwan	VP1	1997	AY382893
P	Pathogenic	China	Rep1; VP1	1988	JF926697
YY	pathogenic	Chian	Full-length	2000	KX000918
FZ91-30	Vaccine	China	Full-length	1991	KT865605

n. a.: not available, but the isolation year is probably close to the 89384 strain

### Rescue of the infectious virus from the pFZ plasmid

The pFZ plasmid was premixed with Lipofectamine 2000 (Invitrogen, Carlsbad, USA) at a ratio of 1:2.5 (μg:μl). The transfection mixture was injected into the chorioallantoic membranes of 11-day–old embryonated goose eggs (0.25 ml per egg, 2.0 μg of plasmid). Simultaneously, transfection of the vector plasmid pBSKN was performed as control.

When the embryos died from viral infection after 48 h post-transfection, their allantoic fluid was pooled. The allantoic fluid was 1:50 diluted in sterile saline and passaged in 11-day-old embryonated goose eggs four times. The presence of the rescued virions was detected by PCR using a pair of primers (5′-GGAACAAACCCAGACTCAAA-3′ and 5′-CCAATCAGCCTATCTTCTACAT-3′) targeting the MDPV VP1 gene. The genome of the rescued virus was further amplified by using the PrimeSTAR Max DNA polymerase (Takara, Dalian, China) after PCR amplification of overlapping viral genome fragments. All amplified fragments were gel-purified and submitted to direct sequencing.

### Introduction of a genetic marker into the rescued virus

To exclude the possibility that the rescued virus originated from contamination during the course of transfection or passaging, a single nucleotide mutation (C → T) was introduced into the Rep1 gene in the pFZ plasmid by overlap PCR. The synonymous nucleotide mutation results in loss of the *Nde*I restriction endonuclease site.

To realize the purpose, two pairs of primers used in the overlap PCR were designed and listed (Table 2). The upstream primer PM-1 and the downstream PM-2 were used to amplify an 839-bp fragment, and the upstream primer PM-3 and the downstream PM-4 were used to amplify a 657-bp fragment. One ng of pFZ plasmid was used as the DNA template in 50 μl of PCR reaction mixture. The amplified fragments were purified with a gel recovery kit (Tiangen, Peiking, China) and dissolved in 30 μl of deionized water. The recovered DNA fragments (1 μl of each fragment) were used as the DNA templates in the subsequent overlap PCR. For overlap PCR, 50 μl of reaction volume also included 25 μl of primeSTAR Max Premix (Takara, Japan), and 20 pmol of each primer (PM-1 and PM-4). The reaction condition was as follows: 95 °C for 1 min followed by 25 cycles of denaturation at 94 °C for 15 s, annealing at 55 °C for 25 s, elongation at 72 °C for 23 s, with a final extension step at 72 °C for 10 min. The 1.5-kb amplicon purified with the gel recovery kit (Tiangen, Peiking, China) was digested with *Nco*I and *Bgl*II and ligated with the linearized pFZ plasmid predigested with the same enzymes.

**Table 2:** Primers used in the overlap PCR to introduce a nucleotide mutation to the rescued virus

Primer name	Primer sequence^a^	Amplification size
PM-1	5′ TCTCC*CCATGG*TTACTCTGG 3′	839 bp
PM-2	5′ GTCCGTAGAGCCATATAGCAT 3′	
PM-3	5′ ATGCTATATGGCTCTACGGAC 3′	657 bp
PM-4	5′ TCTCAGG*AGATCT*CTCTCCGGACT 3′	

^a^The restriction enzyme site (*Nco*I and *Bgl*II) in the primers was denoted with italics and the mutated nucleotide set in primers was underlined

The engineered plasmid pFZ carrying the genetic marker was transfected into 11-day-old embryonated goose eggs to rescue the infectious virus as described above. A 1.5-kb DNA fragment containing the mutation site was amplified by PCR using primer PM-1 and PM-4 from the DNA template extracted from the rescued virions and digested with *Nde*I.

### Biological Characterization of the rescued virus

To study the titer and virulence of the rescued virus, the medium embryos lethal dose (ELD_50_) was calculated by the method of Reed and Muench [30] and mean death time (MDT) was calculated by the method used to evaluate the virulence of Newcastle disease virus [31].

Briefly, the allantoic fluid was diluted in sterile saline to give a tenfold dilution series between 10^-1^ and 10^-5^. For each dilution, 0.2 ml was inoculated into the allantoic cavity of each of five 11-day-old embryonated goose eggs, which were then incubated at 37 °C. The remaining virus dilutions were retained at 4 °C and another five eggs were inoculated with 0.2 ml of each dilution 8 h later and incubated at 37 °C. Each egg was examined twice daily for 10 days and the times of any embryo deaths were recorded. The minimum lethal dose is the highest virus dilution that causes all the embryos inoculated with that dilution to die. MDT is the mean time in hours for the minimum lethal dose to kill all the inoculated embryos.
